# Identification and Validation of Tumor Stromal Immunotype in Patients With Hepatocellular Carcinoma

**DOI:** 10.3389/fonc.2019.00664

**Published:** 2019-08-06

**Authors:** Wei Li, Lin Xu, Jun Han, Kefei Yuan, Hong Wu

**Affiliations:** ^1^Department of Liver Surgery & Liver Transplantation, State Key Laboratory of Biotherapy and Cancer Center, West China Hospital, Sichuan University and Collaborative Innovation Center of Biotherapy, Chengdu, China; ^2^Department of Critical Care Medicine, Sichuan Provincial Hospital for Women and Children, Chengdu, China

**Keywords:** stromal immunotype, hepatocellular carcinoma, prognosis, LASSO COX, TCGA

## Abstract

**Background:** The immune landscape of hepatocellular carcinoma (HCC) is heterogeneous. This study aims to develop the immune type which could improve predictive value of HCC survival.

**Methods:** A total of 208 HCC patients in the testing cohort, 112 patients in the validation cohort and 365 HCC patients in the TCGA database were included in this study. Immune features were assessed by immunohistochemical staining or CIBERSORT method. We constructed prognostic classifiers by LASSO COX analyses in the TCGA cohort, which identified five features out of the 22 types of immune cells.

**Results:** The formulas based on the immunohistochemical staining are as follows: IS_OS_ = 0.648^*^ Macrophage_stromal_ + 0.444^*^Neutrophils_stromal_ + 0.218^*^Tregs_stromal_ – 0.703^*^Memory T cells_stromal;_ IS_DFS_ = 0.285^*^B cells_stromal_ + 0.494^*^Neutrophils_stromal_ + 0.431^*^Tregs_stromal_ – 0.736^*^Memory T cells_stromal_. We classified HCC patients into immune type A subgroup (IS-A) and type B subgroup (IS-B) based on immune scores. The immune type was an independent prognostic indicator for disease-free survival (DFS) and overall survival (OS) in both testing and validation cohorts. Two nomograms (for OS and DFS) that integrated the immune type and clinicopathologic risk factors also showed good predictive accuracy and discriminatory power. IS-A group was correlated with higher immune checkpoint molecule expression. In addition, patients with IS-A and IS-B had distinct mutation signature.

**Conclusion:** The immune types could predict survival and recurrence of HCC effectively. In addition, the immunosuppressive pathways and mutation signature are distinct between two immune types.

## Introduction

Hepatocellular carcinoma (HCC) is the fifth most common cancer and the second most lethal cancer globally (1). Currently, the Barcelona Clinic Liver Cancer (BCLC) classifications and American Joint Committee on Cancer staging system (TNM) remain two systems for routine prognostication and treatment allocation among patients with HCC (2, 3). However, wide variation in clinicopathologic outcomes has been reported among HCC patients with the same tumor stage and received similar treatment regimens (4). Consequently, a new classification for HCC is needed for more precise prediction of long-term survival, thus enabling a more individualized therapeutic schemes with improved survival for HCC patients.

Recently, many publications have demonstrated that tumor-infiltrating lymphocytes in tumors were associated with prognosis (5–9). The type, density, and location of immune cells in tumors had prognostic values that was independent of and superior to those of the traditional TNM stage in some types of cancer (8, 9). An immune score for gastric cancer constructed by Jiang et al. showed a good prognostic value, which were derived from seven immune markers of the tumor, including CD3, CD8, CD45RO, CD45RA, CD57, CD68, and CD66b, and microvascular marker CD34 (8). Fu et al. established a stromal immunotype by measure of five features (Mast cells, macrophages, Tregs, NK cells, and CTLs), which also had a good predictive accuracy in bladder cancer (10). In addition, several studies have utilized immune profiles to identify candidates who might benefit from adjuvant chemotherapy or targeted therapy (11–13). By far, although some studies have illustrated the clinical significance of some immune cells like CD8+ T cells, regulatory T cells (Tregs) and B cells in patients with HCC (14, 15). However, no study has evaluated the whole immune cell landscape of HCC based their prognostic significances.

In the present study, using CIBERSORT (a bioinformatics method), we estimated 22 types of immune cell fraction based on the public HCC cohort from The Cancer Genome Atlas (TCGA) to find out the most relevant prognostic immune cells. Then we performed immunohistochemistry (IHC) to locate these immune cells at tumor tissues of HCC, which aimed to develop an immunotype to predict long-term survival in HCC patients.

## Patients and Methods

### Study Population

The present study was performed after approval by the ethic committee of the West China Hospital, Sichuan University. Our study included three independent cohorts of patients with HCC. The training cohort was from TCGA (http://cancergenome.nih.gov/) database which comprised of 365 HCC patients. We only included patients with available clinical data (including survival data) and mRNA expression data. In addition, we obtained 320 HCC samples at the West China Hospital and patients were enrolled between June 2009 and December 2014. The data of patients undergoing hepatectomy for pathologically proven HCC at our liver surgery center were collected retrospectively and analyzed prospectively. Patients with unavailable tumor samples or clinicopathologic data were excluded. Patients who received previous treatment including transcatheter arterial chemoembolization and radiofrequency ablation were also excluded. The surgical procedure was performed as in our previous study (16). Patients from our hospital were randomly divided into testing cohort (*n* = 208) and validation cohort (*n* = 112) by computer-generated random numbers. The study design is shown in Figure S1. Written consents were acquired from all participants.

Barcelona Clinic Liver Cancer (BCLC) staging system and the American Joint Committee on Cancer (AJCC)-TNM staging system (7th) were utilized to classify patients into different stages. Liver function was evaluated according to the Albumin-Bilirubin (ALBI) grade. Patients were divided into grade 1 and 2 by the cut points of linear predictor and the cut points were set as follows: ALBI grade 1: ≤ −2.60, ALBI grade 2: −2.60 to −1.39 (17). The median follow-up time for the testing cohort was 56.4 and 56.0 months for the validation cohort. In the first 1 year after hepatectomy, patients were followed up at a 2-month interval, and at a 3-month interval thereafter. Overall survival (OS) was defined as the interval between hepatectomy and time of either death or last follow-up. Disease-free survival (DFS) was define as the length of time after liver surgery to tumor recurrence.

### Immunoscore Establishment and Validation

**Figure 1:**
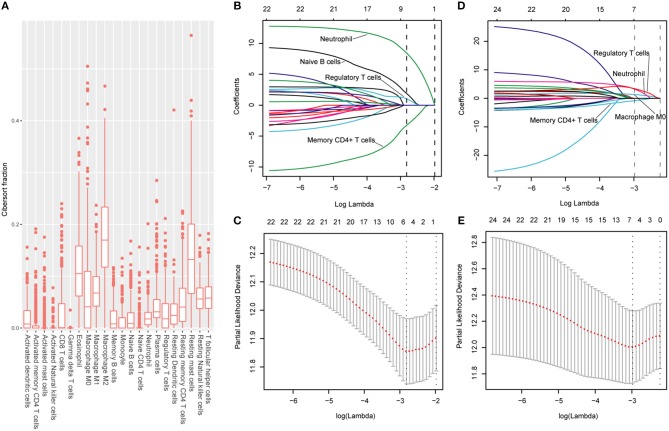
**(A)** Fraction data of the 22 types of immune cells. **(B)** LASSO coefficient profiles of the four selected stromal features for disease-free survival. A dashed vertical line is drawn at the value (logγ = −2.85) chosen by 10-fold cross-validation. **(C)** Partial likelihood deviance of the LASSO coefficient profiles for disease-free survival. **(D)** LASSO coefficient profiles of the four selected stromal features for overall survival. A dashed vertical line is drawn at the value (logγ = −2.9) chosen by 10-fold cross-validation. **(E)** Partial likelihood deviance of the LASSO coefficient profiles for overall survival. The formulas based on the CIBERSORT and LASSO COX are as follows: IS_OS_ = 1.251* Macrophage_stromal_ + 2.795*Neutrophils_stromal_ + 3.111*Tregs_stromal_ – 0.683*Memory T cells_stromal;_ IS_DFS_ = 1.890*B cells_stromal_ + 8.434*Neutrophils_stromal_ + 1.026*Tregs_stromal_ – 3.256*Memory T cells_stromal_. We classified HCC patients into immune type A subgroup (IS-A) and type B subgroup (IS-B) based on cut-off values of immune scores (IS_OS_: 0.21; IS_DFS_: 0.18).

The CIBERSORT (http://cibersort.stanford.edu/) is a well-designed method and has been validated in previous studies on gene expression profiles measured by microarrays (18). LM22 is a signature genes file consisting of 547 genes that accurately discriminate 22 mature human hematopoietic populations and activation states, including seven T cell types, naive and memory B cells, plasma cells, NK cells, and myeloid subsets. The CIBERSORT and LM22 gene signature were utilized to estimate the inferred fractions (for each patient, the sum of estimated fractions for all of the immune cell type equaled 1) of immune cells in the HCC samples. Fractions produced by the CIBERSORT were used to analyze the associations between the survival and 22 human hematopoietic cell phenotypes in TCGA cohort. Fraction data of the 22 types of immune cells are shown in Figure 1A. Least absolute shrinkage and selection operator (LASSO) COX regression analysis was utilized to develop an immunoscore (Figures 1B–E). Finally, five relevant prognostic immune cells for OS (memory T cells, neutrophils, macrophages and regulatory T cells) and DFS (memory T cells, B cells, neutrophils and regulatory T cells) were selected to establish the immune scores.

With Formalin-fixed paraffin-embedded HCC tissues, the tissue microarrays were constructed by standard approaches. Immunohistochemistry (IHC) staining was performed as described in supplementary materials. To validate the types of immune cells selected from the TCGA cohort, we stained the immune cells by IHC with markers as follows: CD45RO (memory T cells), CD66b (neutrophils), CD20 (B cells), CD68 (macrophages), and FOXP3 (regulatory T cells). In addition, the tumors were stained with the following antibodies: CTLA-4, OX40, TIM-3, LAG3, PD-L1, and PD-1. The detailed information about the primary antibodies for IHC staining is shown in Table S1.

### Quantitation of the Stained Immune Cells

Two pathologists who were blinded to the clinical outcome evaluated the outcomes of IHC staining. The density of stained immune cells was assessed in this study. Firstly, after screening the tissue sections at low power (100), we selected three most representative areas of stroma. Next, we evaluated the stained immune cells at 200 magnification and the mean value was adopted (10). The counts of all positive immune cells (CD45RO, CD20, CD66b, CD68, CTLA-4, FOXP3, OX40, LAG3, TIM-3, PD-L1, and PD-1) were changed into cell density (cells/mm^2^). Positive PD-L1 tumor cell staining was defined as more than 1% tumor cells staining on the membrane of the tumor cells.

### Statistical Analysis

Continuous variables were compared by *t*-test or Kruskal-Wallis test and categorical variables were tested by χ2 test or Fisher's exact test. The survival analyses were carried out by the Kaplan-Meier method. Patients were censored at the time of tumor recurrence, death, or end of the study in the log-rank test. The LASSO model was used to select the prognostic markers with statistical significance out of all the 22 types of immune cell features. We established a classifier based on features of multiple immune cells for predicting survival in the TCGA cohort. The “glmnet” R package was utilized to carry out the LASSO COX regression. Restricted cubic spline regression was used to characterize the relationship between immune score and patient survival in all three cohorts (19). Variables which changed HR or β by at least 10% were adjusted, when they were added to or removed from the multivariate models (20). Additionally, clinically clear prognosis-relevant indicators were also included in the adjusted models even they did not confirm to the above condition. Unadjusted analysis without covariates was carried out by univariate COX regression. Variables in the multivariate COX regression analysis included age, sex, HBV infection, HBV-DNA level, ALT and AST level, AFP level, ALBI grade, BCLC stage, tumor differentiation, MVI and immune score. Final Models in multivariate COX analyses for both OS and DFS after stepwise Akaike Information Criterion selection are shown in Table 2. In addition, two nomograms integrating immune type and clinical parameters were constructed according to results of the multivariable models. Based on the identified prognostic factors, the nomograms could be utilized to predict 1-, 3-, and 5-year survival. The discriminative capabilities of the nomograms were assessed by the area under the receiver operating characteristic curve (AUC). Calibration of the model was evaluated graphically by calibration curves. Gene Set Enrichment Analysis (GSEA) analysis was used to identify the pathways that were significantly enriched between stromal immune score type A (IS-A) and stromal immune score type B (IS-B). All statistical analyses were done by R (http://www.R-project.org) and Empower Stats software (www.empowerstats.com, X&Y solutions, Inc. Boston MA).

## Results

### Immunoscore Development and Validation

**Figure 2:**
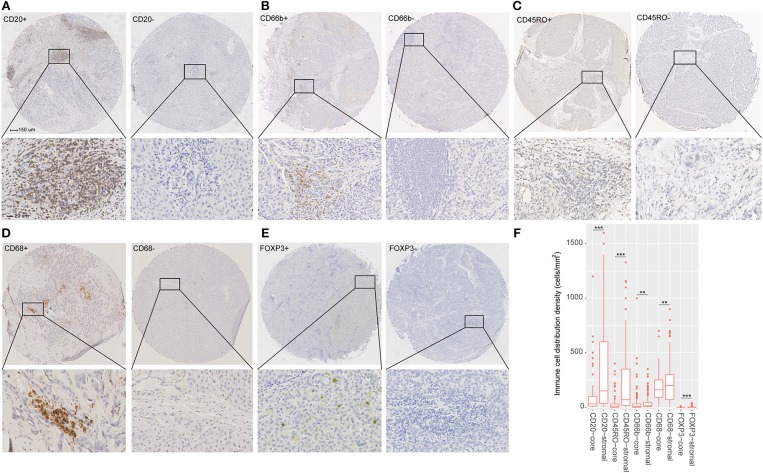
**(A–E)** Tumor infiltration of B cells, neutrophils, Memory T cells, macrophages and regulatory T cells (Tregs) in HCC patients. **(F)** Tumor infiltration density of B cells, neutrophils, Memory T cells, macrophages and Tregs in the total cohort (testing and validation cohorts). The mean values of infiltration density of B cells, neutrophils, Memory T cells, macrophages and Tregs in stroma are 331, 37, 155, 199, and 2 cells/mm^2^. The standard deviation of infiltration density of B cells, neutrophils, Memory T cells, macrophages and Tregs in stroma are 343, 62, 198, 127, and 4 cells/mm^2^. **P < 0.01; ***P < 0.001.

**Figure 3:**
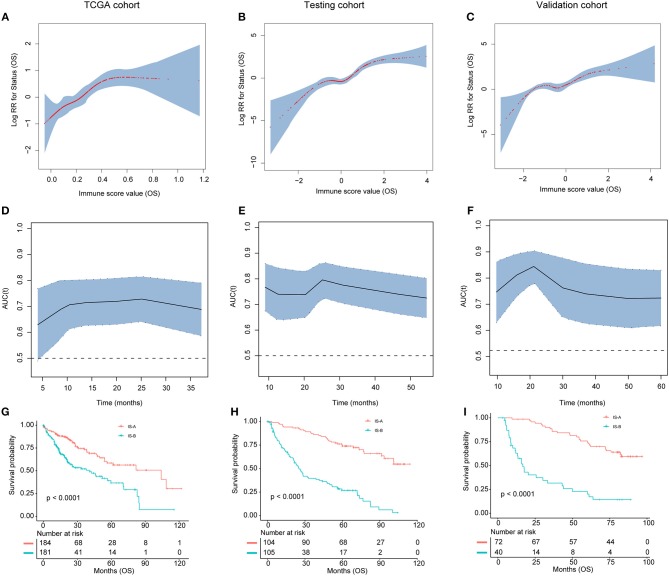
**(A–C)** The restricted cubic spline of the immune score in TCGA, testing, and validation cohorts (OS). **(D–F)**. The immune score had acceptable predictive ability in all three cohorts. OS, overall survival; RR, risk ratio; AUC, area under the receiver operating characteristic curve. **(G–I)** Patients with immune type B (IS-B) had significantly worse overall survival than patients with immune type A (IS-A) in all three cohorts.

The detailed clinicopathologic features of the TCGA, testing, and validation cohorts are shown in Table 1 and Table S2. Specially, patients with IS-B had more cases with MVI in both testing and validation cohorts, which indicated a more aggressive phenotype of IS-B group (Table 1). We built prognostic classifiers by LASSO COX analyses in the TCGA cohort, which identified five features out of the 22 types of immune cells (Figure 1). Notably, number of memory T cells, neutrophils, macrophages, and regulatory T cells (Tregs) were significant prognostic factors for OS, while B cells, memory T cells, neutrophils, and Tregs were prognostic factors for DFS. Figure S2 shows the restricted cubic spline functions of the immune cells in the TCGA, testing and validation cohorts. Next we stained memory T cells, neutrophils, macrophages, Tregs and B cells, respectively. The representative images of these immune cells are shown in Figures 2A–E. As shown in Figure 2F, all five types of immune cells were significantly more abundant in the stroma (both testing and validation cohorts). Meanwhile, we found that stromal immune cell infiltration was significantly relevant to HCC patient prognosis compared with infiltration in tumor core (Table S3), thus, we used stromal immune cells (Z score of expressions) to construct the immune index. In the testing cohort, by multivariable COX model, we calculated the coefficients of the formulas. The formulas are as follows: IS_OS_ = 0.648^*^Macrophage_stromal_ + 0.444^*^Neutrophils_stromal_ + 0.218^*^Tregs_stromal_ – 0.703^*^Memory T cells_stromal;_ IS_DFS_ = 0.285^*^B cells_stromal_ + 0.494^*^Neutrophils_stromal_ + 0.431^*^Tregs_stromal_ – 0.736^*^Memory T cells_stromal._ Using X-tile plots, we classified patients into type A (IS-A) and type B (IS-B) groups based on the cut-off values of immune score (0.1 for OS; 0.13 for DFS). Restrictive cubic spline functions of immune scores in the TCGA, testing and validation sets showed that both scores (for OS and DFS) presented linear profiles (Figures 3A–C and Figures S3A–C).

**Table 1:** Clinicopathological characteristics of patients according to the immunotype (OS) in the testing and validation Cohorts.

**Variable**	**Testing cohort (*****n*** **=** **208)**			**Validation cohort (*****n*** **=** **112)**		
	**IS-A**	**IS-B**	***P***	**IS-A**	**IS-B**	***P***
Age, years	51.5 ± 11.8	50.4 ± 12.8	0.518	52.5 ± 13.3	45.5 ± 13.3	0.009
Gender			0.599			0.548
Male	89 (86.4%)	88 (83.8%)		54 (75.0%)	32 (80.0%)	
Female	14 (13.6%)	17 (16.2%)		18 (25.0%)	8 (20.0%)	
HBV infection			0.687			0.016
Negative	10 (9.7%)	12 (11.4%)		17 (23.6%)	2 (5.0%)	
Positive	93 (90.3%)	93 (88.6%)		55 (76.4%)	38 (95.0%)	
HBV-DNA, IU/mL			0.255			0.445
<103	36 (44.4%)	27 (35.5%)		20 (44.4%)	8 (33.3%)	
≥103	45 (55.6%)	49 (64.5%)		25 (55.6%)	16 (66.7%)	
AFP, ng/mL			0.248			0.819
<400	65 (63.1%)	58 (55.2%)		43 (59.7%)	23 (57.5%)	
≥400	38 (36.9%)	47 (44.8%)		29 (40.3%)	17 (42.5%)	
Preoperative ALT, IU/L	49.7 ± 38.1	50.4 ± 42.9	0.893	43.8 ± 40.2	44.4 ± 25.3	0.928
Preoperative AST, IU/L	44.8 ± 28.7	53.2 ± 36.0	0.067	44.7 ± 29.5	49.9 ± 27.4	0.359
ALBI Grade 1/2			0.278			0.277
Grade 1	73 (70.9%)	67 (63.8%)		57 (79.2%)	28 (70.0%)	
Grade 2	30 (29.1%)	38 (36.2%)		15 (20.8%)	12 (30.0%)	
Tumor number			0.323			0.659
Single	86 (83.5%)	82 (78.1%)		60 (83.3%)	32 (80.0%)	
Multiple	17 (16.5%)	23 (21.9%)		12 (16.7%)	8 (20.0%)	
Tumor size, cm	5.6 ± 3.5	6.6 ± 3.8	0.086	5.1 ± 2.8	6.1 ± 3.1	0.132
AJCC-TNM Stage			0.086			0.206
Stage I	54 (52.4%)	39 (37.1%)		41 (56.9%)	21 (52.5%)	
Stage II	26 (25.2%)	35 (33.3%)		12 (16.7%)	12 (30.0%)	
≥ Stage III	23 (22.3%)	31 (29.5%)		19 (26.4%)	7 (17.5%)	
BCLC Classification			0.096			0.491
A	84 (81.6%)	78 (74.3%)		59 (81.9%)	30 (75.0%)	
B	17 (16.5%)	18 (17.1%)		11 (15.3%)	7 (17.5%)	
C	0 (0%)	0 (0%)		2 (2.8%)	3 (7.5%)	
Tumor differentiation			0.064			0.152
Good	67 (65.0%)	55 (52.4%)		46 (63.9%)	20 (50.0%)	
Poor	36 (35.0%)	50 (47.6%)		26 (36.1%)	20 (50.0%)	
MVI			0.031			0.023
No	70 (68.0%)	56 (53.3%)		59 (81.9%)	25 (62.5%)	
Yes	33 (32.0%)	49 (46.7%)		13 (18.1%)	15 (37.5%)	

*HBV, hepatitis B virus; AFP, alpha fetoprotein; ALT, alanine aminotransferase; AST, aspartate aminotransferase; ALBI, albumin-bilirubin; FIB-4, Fibrosis 4 Score; AJCC, American Joint Committee on Cancer; BCLC, Barcelona Clinic Liver Cancer; MVI, microvascular invasion*.

### Association of Stromal Immune Type With Patient Survival

We evaluated the distribution of immune scores, survival status, and the expression level of the immune features in the TCGA (Figure S4), testing and validation cohorts (Figure S5). For OS, tumors with high immune score generally exhibited increased CD66b, CD68, and FOXP3 expression and reduced CD45RO expression. For DFS, tumors with high immune score generally showed increased CD66b, CD20, and FOXP3 expression and reduced CD45RO expression. Patients with high immune score had more recurrences and deaths.

As shown in Figures 3G–I and Figures S3G–I, patients in IS-B group showed significantly worse OS and DFS than those in the IS-A group in all three cohorts. In the testing cohort, the 1-, 3-, and 5-year DFS rates for IS-A and IS-B were 88.0, 75.6, and 65.9%; 42.6, 25.1, and 20.3%, respectively. The 1-, 3-, and 5-year OS rates were 95.2, 87.2, and 74.0% for IS-A, and 72.2, 39.6, and 26.8% for IS-B, respectively. Univariate COX regression analysis identified stromal immune type was a statistically significant factor associated with OS and DFS (Table 2 and Figure S4) in both testing and validation cohorts. In the present study, we calculated AUC to confirm the predictive accuracy of the immune score. As shown in Figures 3D–F and Figures S3D–F, the immune score had acceptable predictive ability in all three cohorts.

**Table 2:** Univariate and multivariate analysis of the testing cohort.

**Variable**	**Univariate analysis**			**Multivariate analysis**		
	**HR**	**95%CI**	***P***	**HR**	**95%CI**	***P***
**Overall survival**						
Age, years	1.00	0.99–1.02	0.698	1.02	1.00–1.03	0.045
Gender, female vs. male	0.74	0.41–1.35	0.325			
HBV, positive vs. negative	1.63	0.79–3.34	0.186	2.31	1.07–4.97	0.032
HBV-DNA, >103/ ≤ 103 IU/mL	1.97	1.19–3.27	0.008			
AFP, ≥400 ng/mL vs. < 400 ng/mL	1.04	0.71–1.53	0.825			
Preoperative ALT, IU/L	1.00	1.00-1.01	0.048			
Preoperative AST, IU/L	1.01	1.00–1.01	0.021			
ALBI Grade, Grade 2 vs. Grade 1	1.50	1.03–2.21	0.037			
BCLC Classification						
BCLC B-C vs. BCLC A	2.11	1.34–3.32	0.001	2.05	1.26–3.32	0.004
Tumor differentiation, poor vs. good	1.16	0.80–1.69	0.438			
MVI, yes vs. no	1.97	1.35–2.86	<0.001	1.67	1.12–2.48	0.012
Immune score, IS-B vs. IS-A	5.14	3.37–7.85	<0.001	5.30	3.40–8.25	<0.001
**Disease-free survival**						
Age, years	1.00	0.99–1.02	0.956			
Gender, male vs. female	1.23	0.74–2.05	0.419			
HBV, positive vs. negative	1.36	0.69–2.69	0.380			
HBV-DNA, IU/mL, >103/ ≤ 103	1.52	0.96–2.43	0.077			
AFP, ≥400 ng/mL vs. <400 ng/mL	1.71	1.17–2.50	0.006	1.42	0.94–2.13	0.095
Preoperative ALT, IU/L	1.00	1.00–1.01	0.403			
Preoperative AST, IU/L	1.00	1.00–1.01	0.117			
ALBI Grade, Grade 2 vs. Grade 1	1.23	0.82–1.83	0.323			
BCLC Classification						
BCLC-B vs. BCLC-A	1.79	1.10–2.90	0.018	1.53	0.91–2.55	0.107
BCLC-C vs. BCLC-A	2.94	1.42–6.10	0.004	2.15	0.99–4.66	0.053
Tumor differentiation, poor vs. good	1.33	0.91–1.95	0.139			
MVI, yes vs. no	1.91	1.31–2.81	<0.001	1.42	0.93–2.16	0.102
Immune score, IS-B vs. IS-A	4.21	2.79–6.37	<0.001	3.24	2.13–4.94	<0.001

*HBV, hepatitis B virus; AFP, alpha fetoprotein; ALT, alanine aminotransferase; AST, aspartate aminotransferase; ALBI, albumin-bilirubin; FIB-4, Fibrosis 4 Score; AJCC, American Joint Committee on Cancer; BCLC, Barcelona Clinic Liver Cancer; MVI, microvascular invasion; HR, hazard ratio; CI, confidence interval*.

In multivariate COX analyses, immune type was also observed to be an independent prognostic indicator for both OS and DFS in the TCGA, testing and validation groups (Table 2, Tables S4, S5).

### Construction of the Nomograms

**Figure 4:**
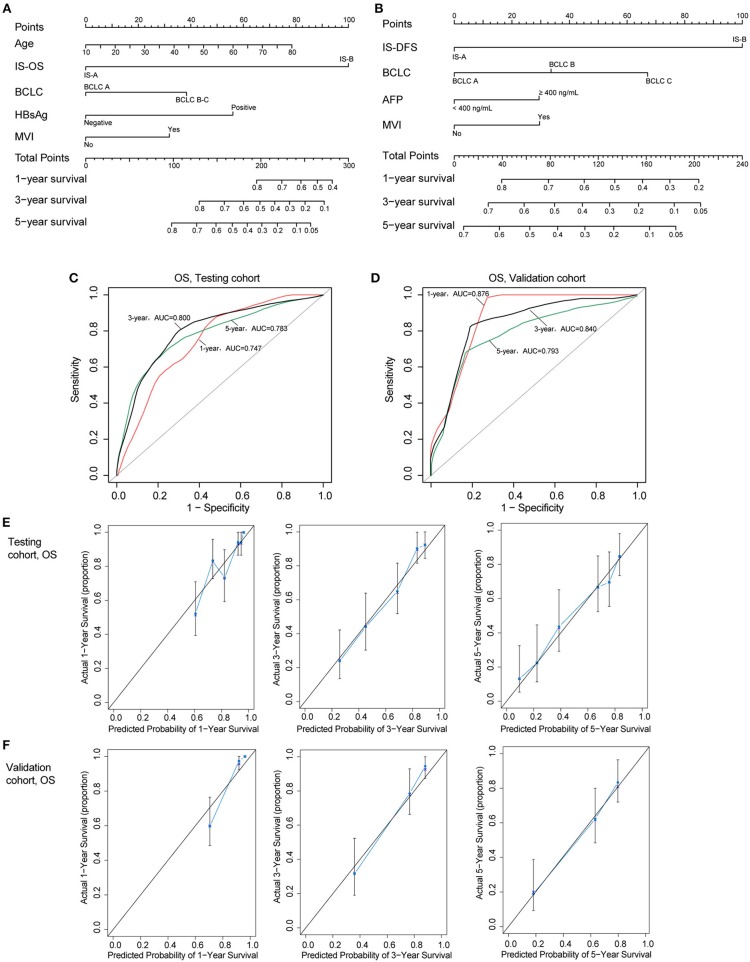
**(A,B)** Nomograms (for OS and DFS) that integrated the immune type and clinicopathologic risk factors. To calculate the probability of status, sum up the points identified on the scale for all the variables and draw a vertical line from the total points scale to the probability scale. **(C,D)** ROC curves showing the predictive accuracy (1-, 3-, 5-year AUC) of the nomogram for OS in testing and validation cohorts. **(E,F)** Calibration curves showing the discriminatory power of the nomogram for OS in testing and validation cohorts.

Two nomograms (for OS and DFS) that integrated the immune type and clinicopathologic risk variables were constructed, which could provide a clinically useful quantitative method to predict the probability of 1-, 3-, and 5-year OS and DFS in patients with HCC (Figures 4A,B; Table 2). The predictive ability of the nomogram for OS in testing and validation cohorts is shown in Figures 4C,D (1-, 3-, 5-year AUC). Calibration plots for the probability of 1, 3, and 5 year survival also showed good agreement between the predictions and the observations in both cohorts (Figures 4E,F). The ROC and calibration curves for DFS in the testing and validation cohorts are shown in Figure S6. Our nomograms also showed better predictive accuracy in both cohorts compared to the TNM (7^th^) and BCLC classification (Figure S7).

### Identification of Stromal Immunotype Associated Biological Pathways and Immune Checkpoint Molecules

**Figure 5:**
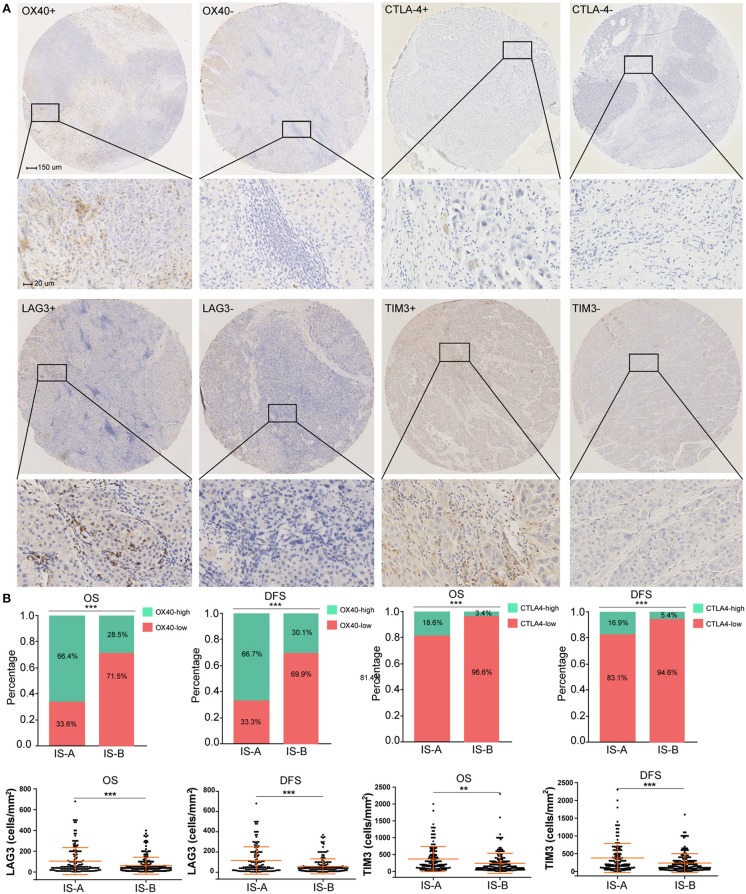
Higher expression of multiple immunomarkers in IS-A group. **(A)** Representative immunohistochemistry images (positive markers and negative control) of OX40, CTLA-4, LAG3, and TIM-3 in HCC. The bars (150 and 20 μm) are shown in the upper left figures. **(B)** Associations of immune type with the immune check-point markers. According to the data distributions, the optimal cut-off values for OX40 and CTLA-4 were selected to perform comparisons between groups. TIM-3 and LAG3 expressions between two immune types were compared by continuous data. **P < 0.01; ***P < 0.001.

The GSEA analyses showed that IS-A subgroup (immunotype based on both OS and DFS) was highly enriched in natural killer cell mediated cytotoxicity, T cell receptor signaling and antigen processing and presentation pathways (Figure S8). In addition, by IHC staining, we observed that the expression of several immune checkpoint or co-stimulatory molecules (OX40, CTLA-4, LAG3, TIM-3, immune cell PD-L1, PD-1) was significantly higher in IS-A subgroup (Figure 5 and Figure S9), whereas we found no association between the PD-L1 (tumor cell) level and immune type.

### Immunotype-Related Mutational Events

**Figure 6:**
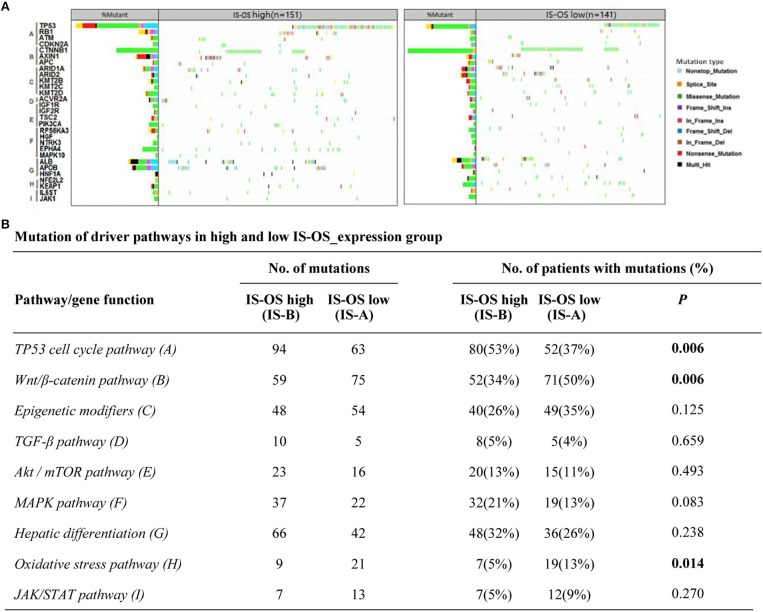
Pathway-based gene mutations in IS-A and IS-B samples (overall survival). **(A)** Waterfall plot of mutated genes in IS-A and IS-B samples. Gene mutations were ordered by distinct pathways: A indicates the TP53 cell cycle pathway; B, the Wnt/β-catenin pathway; C, epigenetic modifiers; D, TGF-β signaling; E, the Akt/mTOR pathway; F, the MAPK pathway; G, hepatic differentiation; H, the oxidative stress pathway; and I, JAK/STAT signaling. Each column represents a sample. Boxes with different colors indicate different types of non-synonymous mutations. **(B)** Numbers of mutations and numbers of patients with or without mutations in IS-A and IS-B groups.

Previous researches have showed the landscape of mutations in HCC driver genes and identified major mutational pathways that were engaged in HCC. Therefore, using the TCGA data, we explored whether our immune type is correlated with mutated genes and relative eight pathways (frequently mutated in HCC). Finally, for immune type based on OS, the percentage of patients with TP53 cell cycle pathway mutations in the IS-A group (37%) was significantly lower than that in the IS-B group (53%) (*P* = 0.006). In addition, the percentage of patients in the IS-A group (50%) with mutations in Wnt/β-catenin signaling was significantly higher than that of the IS-B group (34%) (*P* = 0.006; Figure 6). In addition, more patients in IS-A group had oxidative stress pathway mutations than those in IS-B group (13% vs. 5%; *P* = 0.014). The trends were also observed for immune type based on DFS, however, no statistically significances were observed (Figure S10). In conclusion, these data suggest that IS-A and IS-B HCCs might have distinct mutated driver genes and pathways.

## Discussion

Accurate risk stratification and long-term prognostic prediction is essential to properly select suitable therapeutic modality for patients with HCC. Integrating diverse independent prognostic variables into a single formula could improve the prognostic ability significantly (9). In this study, we constructed and validated two immune scores (for OS and DFS, respectively) to improve survival prediction for HCC. As novel prognostic tools, our immune scores comprised features of five types of immune cells in HCC. The results showed clear separations of overall and disease-free survival curves between patients with high (IS-B) and low (IS-A) immunoscores.

Recently, immune profiling studies have gained a forefront position in cancer researches. Several studies based on immunoscores have been published to describe the immune landscape and to provide independent prognostic models for survival prediction in patients with several types of solid tumor, including HCC (6, 8, 9). In addition, previous data also indicated that specific immune cells were highly associated with treatment responses (e.g., chemotherapy and immune-modulating therapies) (7). However, previous studies have established many molecular signatures (include genes, microRNAs, lncRNAs, and epigenetic biomarkers) to predict long-term survival in patients with tumor (6, 21, 22). These signatures failed to be widely used clinically, as the variability of measurements in gene sequencings, inconsistencies in assay platforms, and the requirement for specialized analyses. In contrast to other studies evaluating immune cells in HCC, our immune type was firstly developed based on a high-throughput gene expression profile generated using the newly developed algorithm CIBERSORT (23). Then we validated our immunoscores by IHC staining, which potentially can been widely applied in clinical practice. In addition, HCC is a clinically heterogeneous disease with large variations in the clinical outcomes. In this study, the novel immune type comprising five selected immune features of HCC was developed, as a prognostic tool independent of other clinical and pathological features.

Notably, we constructed two immune scores for OS and DFS, respectively. In comparison, previous studies usually utilized one formula to estimate these two different endpoints (8–10). However, the immune responses associated with OS and DFS might be different. We found that the IS-B subgroup consisted of a Neutrophil^high^Macrophage^high^Treg^hig^Memory T-cell^low^ group associated with a worse OS, whereas a Neutrophil^high^B-cell^high^Treg^hig^Memory T-cell^low^ group correlated with a worse DFS. In this study, we have linked neutrophils, macrophages and Tregs to poor OS and DFS, which is in accordance with several studies in HCC (6, 15, 24, 25). In contrast, memory T cells was associated with a prolonged DFS and OS. This is in line with recent findings from Jiang et al., who reported that memory T-cell was one of the most statistically significant favorable prognostic immune cell populations for gastric cancer (9). Interestingly, CD8+ CTL cells failed to be a favorable prognostic factor in HCC. In patients with HCC, we observed that a higher number of B cells was related to a worse DFS. This is of particular interest in light of the current controversy of the role of B cells in tumors. Previous studies have demonstrated both anti- and pro-tumoral effects and prognostic studies have also linked B-cells to increased and decreased survival (14, 26–29). The tumor promoting function involves regulation of the T-cell-mediated tumor cell killing function and responses to chemotherapy, or secretion of pro-tumoral stimulatory cytokines (30–32). Further studies should be performed to explore the independent role of B cells in the long-term prognosis of HCC patients.

In this study, we found that IS-A was correlated with higher immune checkpoint molecule expression. These data suggested that our immunotype might also be used as a predictor of upcoming popularity of immunotherapy in HCC. Patients with IS-A had diverse immunosuppressive mechanisms and indicated that combinations of targeted therapies may be effective in HCC treatment. The analysis of high-throughput sequencing data in the TCGA database showed that IS-A and IS-B (immunotype based on OS) had distinct mutation signature. Spranger et al. demonstrated that activated β-catenin signaling pathway could impair the anti-tumor immune response by defecting the recruitment of dendritic cells to the tumor microenvironment (33). Molecular analysis of the human metastatic melanoma tissues observed an association between activation of the WNT/β-catenin signaling pathway and absence of a T-cell gene expression profile (33, 34). However, Malladi et al. showed that WNT signaling silence can lead to tumor cell quiescence and evade innate immunity to remain latent for extended periods (35). All these data made this issue complicated and controversial. In the present study, we found that IS-B subgroup (memory T cells low) had less patients with Wnt/β-catenin mutation, suggesting an immunosuppressive tumor microenvironment with decreased activity of the Wnt/β-catenin pathway. In addition, loss or mutation of TP53 has also been linked to alterations in anti-tumor immunity (poor tumor immunogenicity and ultimately immune escape) as well as dysregulation of cell cycle and apoptosis (36, 37). In line with this notion, our results showed that IS-B subgroup had more patients with TP53 pathway mutation.

This study had several limitations. First, it was retrospective in nature and all specimens were obtained from patients in the West China Hospital. Therefore, our results need to be validated in a prospective and larger cohorts. Second, in this study, the underlying biologic mechanisms behind the relationship of immune type and patient prognosis were not clearly investigated, and further studies may provide more details for better understanding of the role of the immune profile in the development and invasion of HCC. Third, we selected several types of immune cells by CIBERSORT method instead of assessing them all, thus other important immune phenotypes may be missed. Finally, it is unclear whether the immune score could be applied in samples from liver biopsies. Future studies should be carried out to illustrate this issue.

In conclusion, we have characterized the immune cell infiltration in HCC with three independent cohorts. We defined two immune types by integrating the indicators of the immune cell infiltration in HCC. The immune type could be used as a prognostic and predictive tool to identify HCC patients with different long-term survival. The immune types might have significant implications for the personalized follow-up after surgery and decision-making regarding individualized therapies.

## Data Availability

The raw data supporting the conclusions of this manuscript will be made available by the authors, without undue reservation, to any qualified researcher.

## Ethics Statement

All procedures performed in studies involving human participants were in accordance with the ethical standards of the institutional and/or national research committee and with the 1964 Helsinki declaration and its later amendments or comparable ethical standards.

## Author Contributions

HW proposed the study. WL, KY, and JH performed the research and wrote the first draft. WL collected and analyzed the data. HW is the guarantor. LX preformed the IHC staining. WL, LX, JH, KY, and HW contributed to the design and interpretation of the study, and to further drafts. WL, LX, JH, KY, and HW have read and approved the final version to be published.

### Conflict of Interest Statement

The authors declare that the research was conducted in the absence of any commercial or financial relationships that could be construed as a potential conflict of interest. The reviewer MS and handling editor declared their shared affiliation.
