# Supplementary material for: Identification and Validation of Tumor Stromal Immunotype in Patients With Hepatocellular Carcinoma
Source: Front Oncol. 2019 Aug 6;9:664. doi: 10.3389/fonc.2019.00664 (PMC6691778; doi:10.3389/fonc.2019.00664)
Supplement: Supplementary file 1 [file Data_Sheet_1.docx]

**Identification and validation of stromal immunotype in patients with hepatocellular carcinoma**

**Wei Li, Lin Xu, Jun Han, Kefei Yuan, Hong Wu**

**Author Affiliations:**

Department of Liver Surgery & Liver Transplantation Center, West China Hospital, Sichuan University, Chengdu 610041, China

**Correspondence**

Hong Wu, Department of Liver Surgery and Liver Transplantation Centre, West China Hospital, Sichuan

University, Chengdu 610041, Sichuan Province, China. E-mail: [wuhong7801@163.com](mailto:wuhong7801@163.com)

**Conflict of interest:** No benefits in any form have been received or will be received from a commercial party related directly or indirectly to the subject of this article.

| **Table of contents** |
| --- |
| **2 Supplementary Methods**  **3-12 Supplementary Figures**  **12-17 Supplementary Tables** |

**Methods**

**Immunohistochemistry**

Immunohistochemistry was performed according to standard protocols. Sections were baked at 60 ℃ for 40 minutes, de-waxed in xylene, and rehydrated in decreasing concentrations of ethanol. Prior to staining, the sections were subjected to endogenous peroxidase blocking in 3% of H_2_O_2_ solution in methanol for 15 min. Antigen retrieval was carried out by heating in a microwave for 15 min in citrate antigen retrieval solution (PH: 6). After incubated with monoclonal antibodies against CD20, CD45RO, CD66b, CD68, FOXP3, PD-1, PD-L1, TIM-3, LAG3, OX40 and CTLA-4 overnight at 4°C and then incubated with a labeled polymer/HRP amplification system (ZLI-9018 and PV-6000, ZSGB-BIO, China) for 30 min. The immunoreaction was detected after treatment with diaminobenzidine chromogen for 1 minutes. All staining runs included a no-primary-antibody control. The antibody dilutions and antigen retrieval are shown in Table S1. Immunoreaction images were viewed and captured by the Image-Pro-Plus 6.0 software.

Fig. S1 Study design of this study. Patients in the TCGA cohort were used as the training cohort. By CIBERSORT method and LM22 gene signatures, five immune features were selected as the prognosis relevant features. To validate the results observed in the TCGA cohort, immunohistochemistry staining of these five immune markers was performed for patients in the testing and validation cohorts.


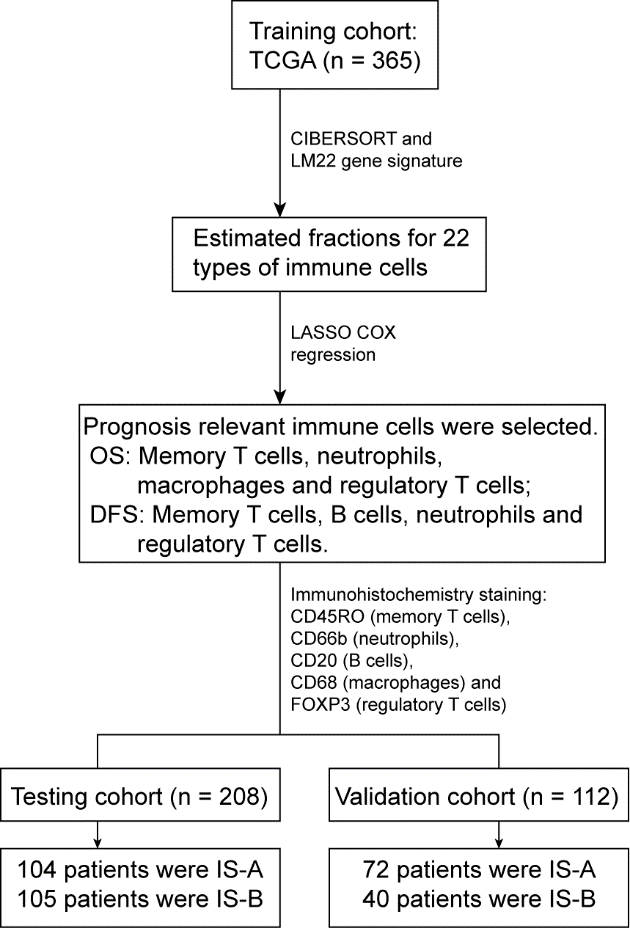


Fig. S2 The restricted cubic spine functions of the selected immune cells in the TCGA, training and validation cohorts.


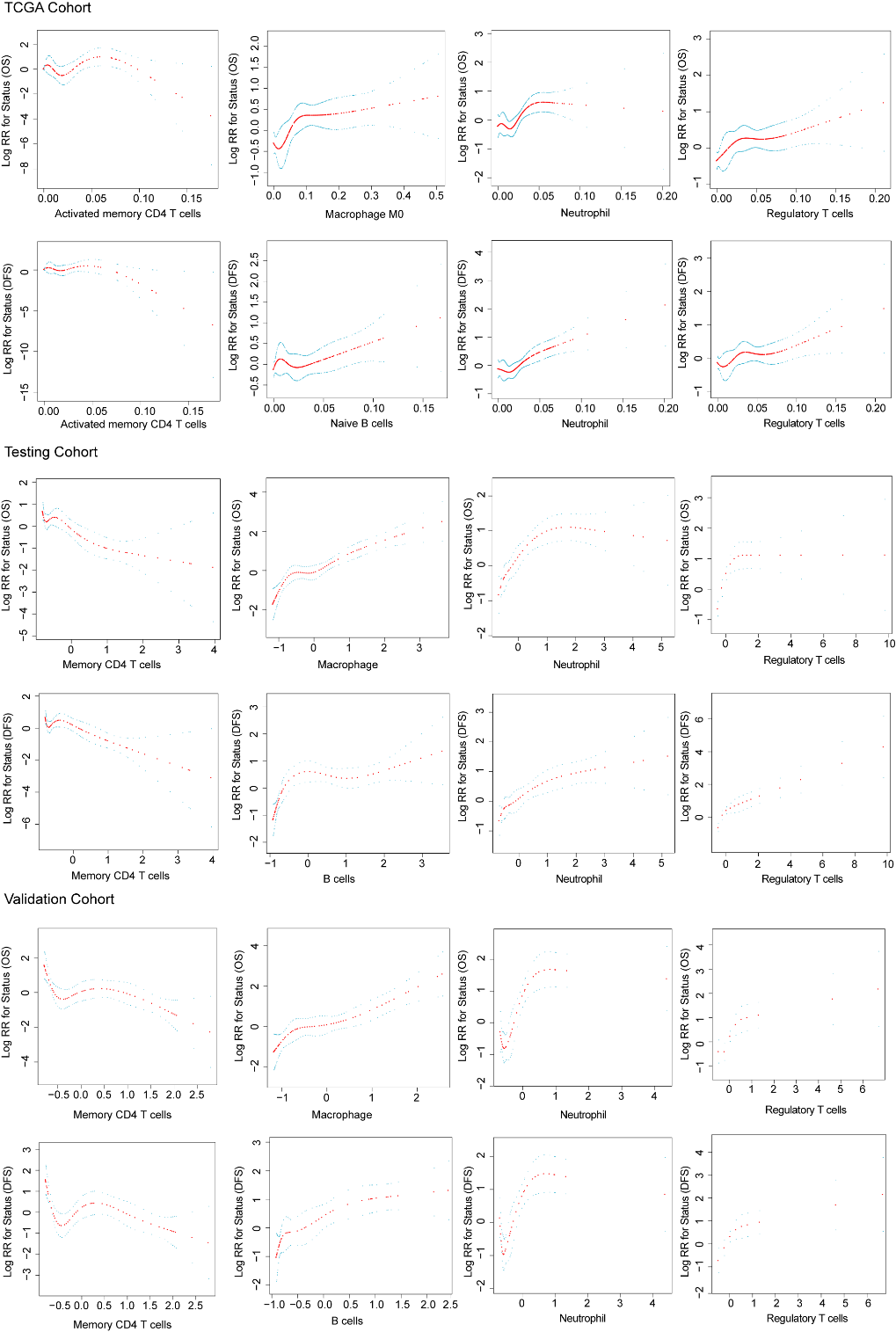


Fig. S3 (A-C) The restricted cubic spine of the immune score in TCGA, testing and validation cohorts (DFS). (D-F): The immune score had acceptable predictive ability in all three cohorts. DFS, disease-free survival; RR, risk ratio; AUC, area under the receiver operating characteristic curve. (G-I) Patients with immune type B (IS-B) had significantly worse disease-free survival than patients with immune type A (IS-A) in all three cohorts.


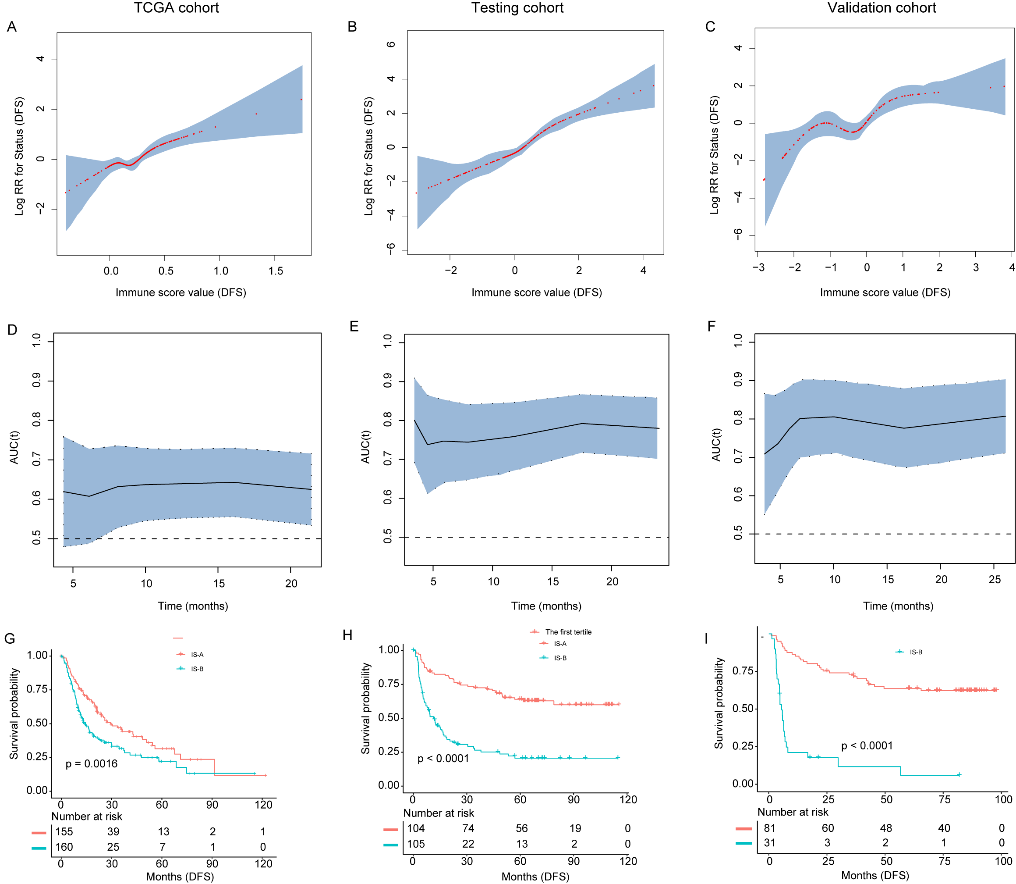


Fig. S4 The distribution of immune scores, survival statuses, and the expression of the immune features in the TCGA cohort. OS, overall survival; DFS, disease-free survival.


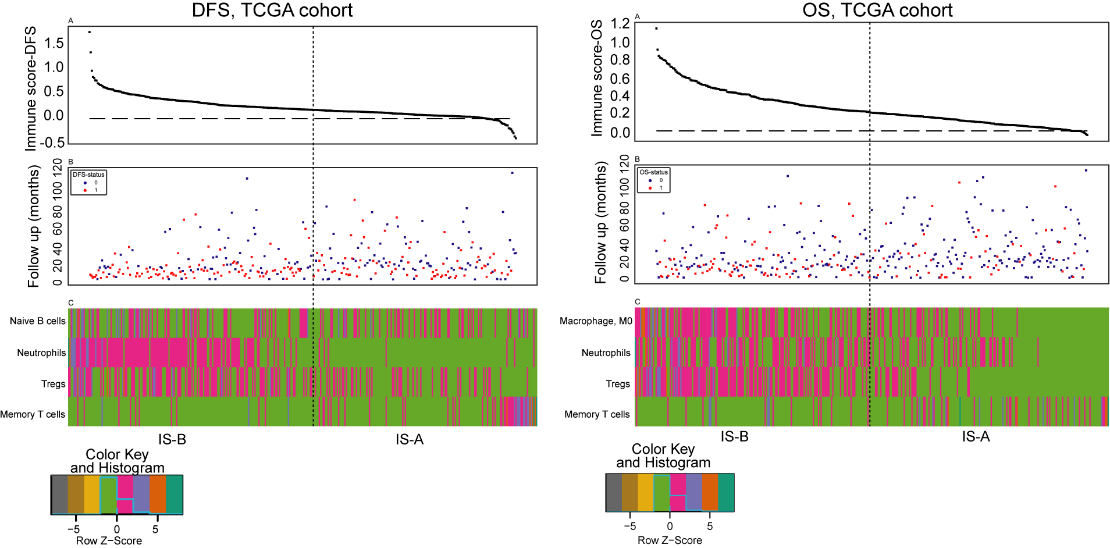


Fig. S5 The distribution of immune scores, survival statuses, and the expression of the immune features in the testing and validation cohorts. OS, overall survival; DFS, disease-free survival.


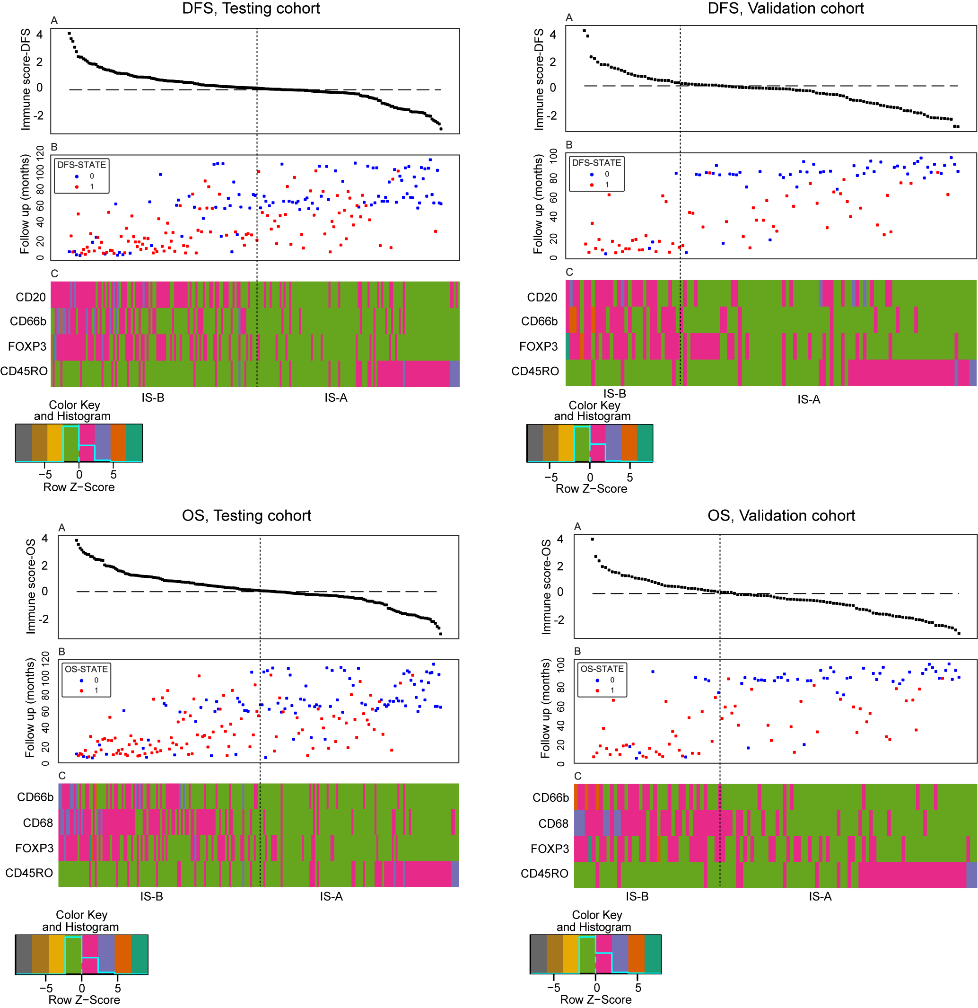


Fig. S6 (A-B) The ROC and calibration curves for disease-free survival in the testing and validation cohorts. DFS, disease-free survival.


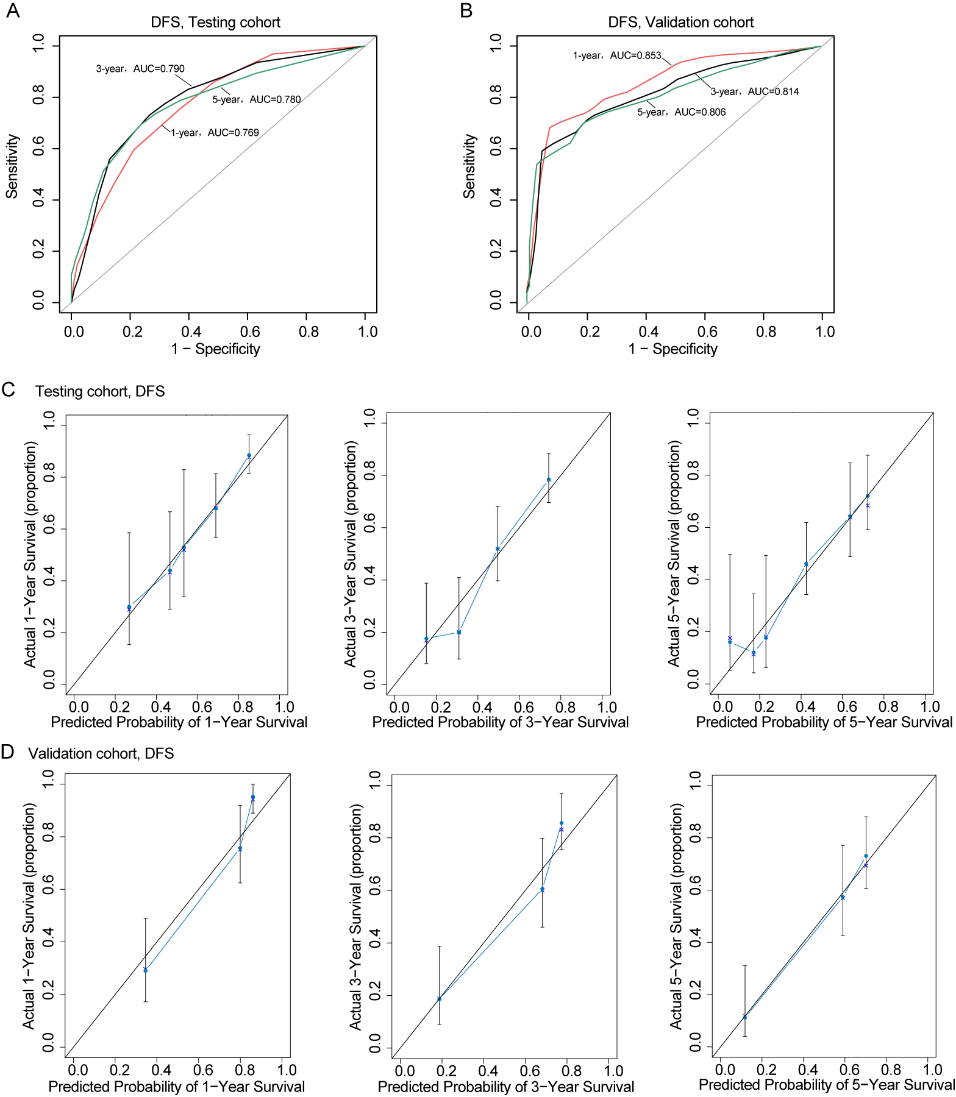


Fig. S7 ROC curves of the TNM (7^th^) and BCLC classification for overall and disease-free survival in the testing and validation cohorts. OS, overall survival; DFS, disease-free survival.


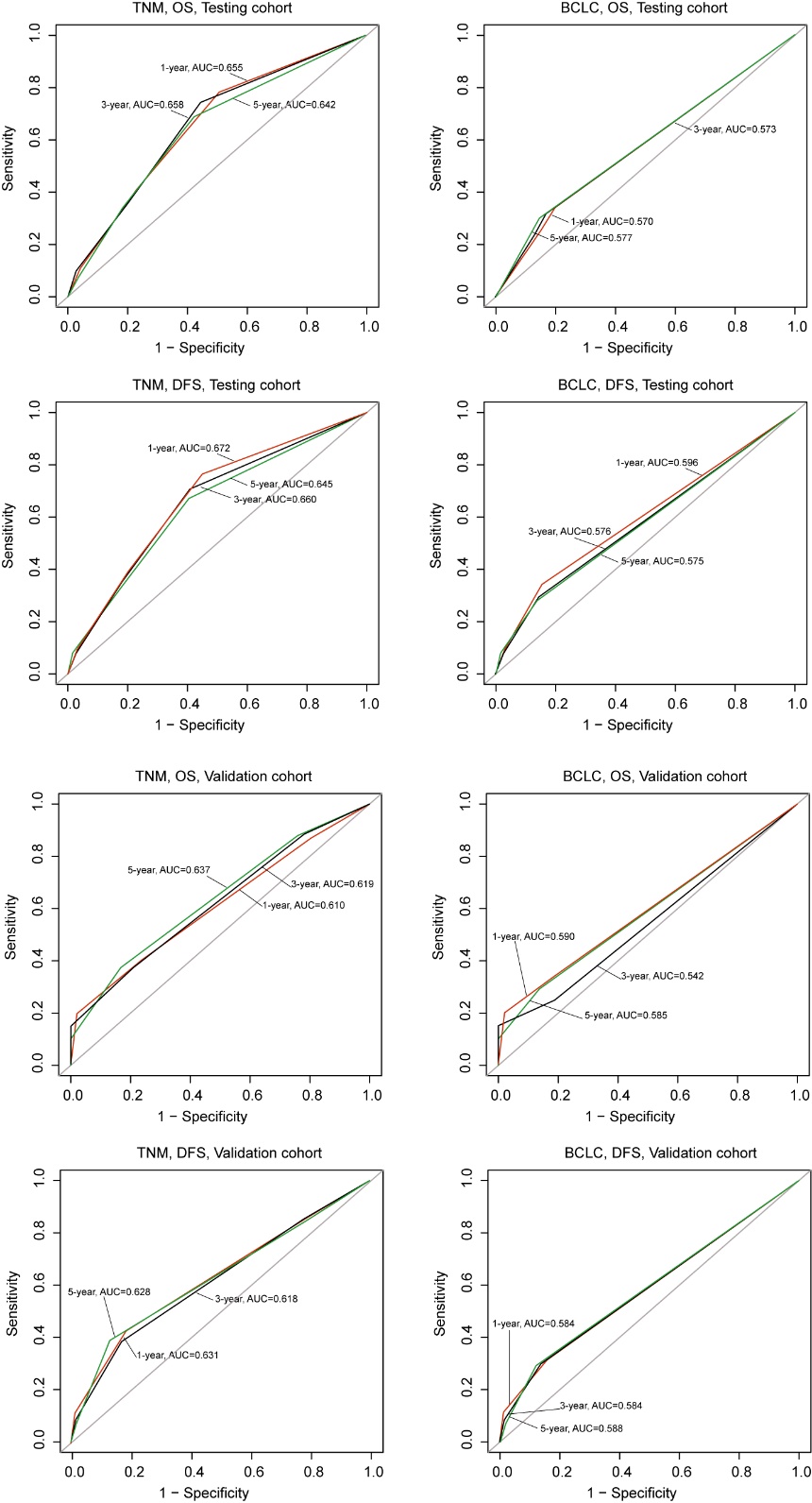


Fig. S8 The GSEA analyses showed that IS-A subgroup (immunotype based on both OS and DFS) was highly enriched in natural killer cell mediated cytotoxicity, T cell receptor signaling and antigen processing and presentation pathways. OS, overall survival; DFS, disease-free survival.


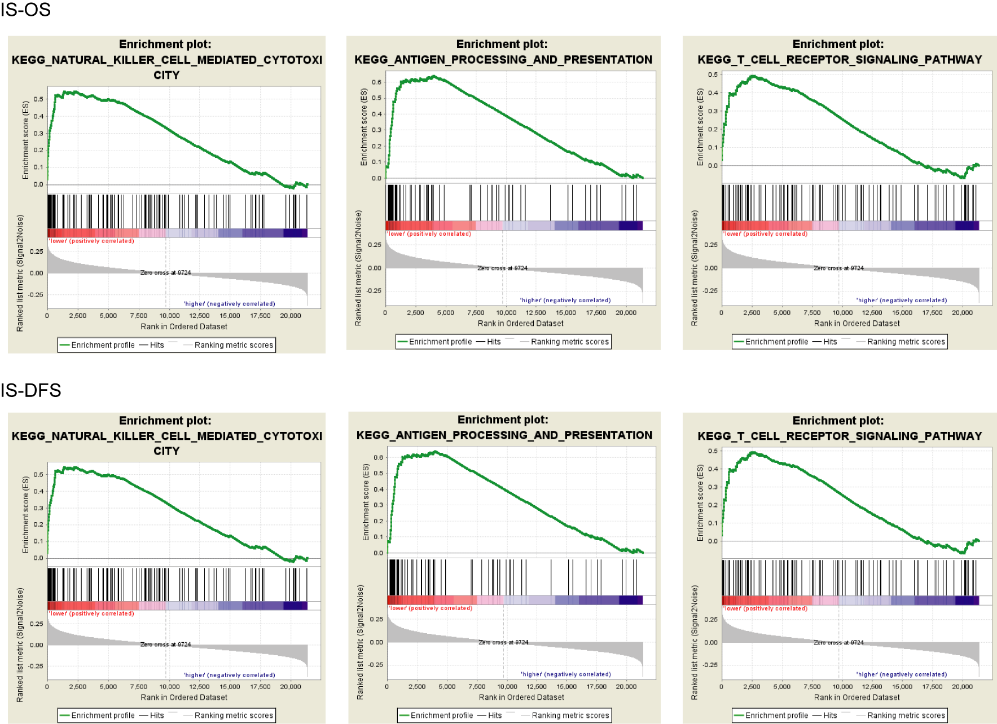


Fig. S9 (A-C): Representative immunohistochemistry images (positive markers and negative control) of PD-1 and PD-L1 in HCC. The bars (150 µm and 20 µm) were shown in the upper left figures. B: Associations of immune type with the markers. (D) According to the data distributions, the optimal cut-off values for PD-L1 (tumor cell) and PD-1 were selected to perform comparison between groups. Positive PD-L1 tumor cells staining was defined as more than 1% tumors cells staining on the membrane of the tumor cells. (E) The PD-L1 (immune cell) were compared by continuous data between two types.


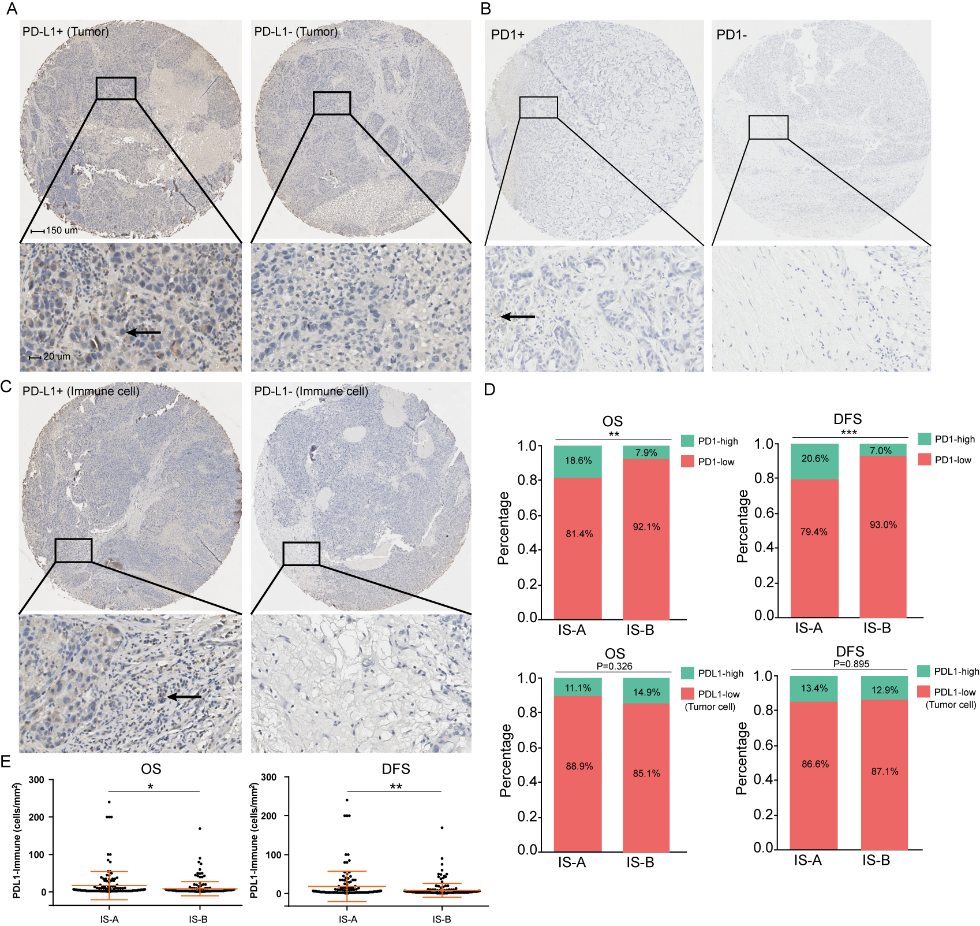


Fig. S10 Pathway-based gene mutations in IS-A and IS-B samples (disease-free survival). (A) Waterfall plot of mutated genes in IS-A and IS-B samples. Gene mutations were ordered by distinct pathways: A indicates the TP53 cell cycle pathway; B, the Wnt/β-catenin pathway; C, epigenetic modifiers; D, TGF-β signaling; E, the Akt/mTOR pathway; F, the MAPK pathway; G, hepatic differentiation; H, the oxidative stress pathway; and I, JAK/STAT signaling. Each column represents a sample. Boxes with different colors indicate different types of non-synonymous mutations. (B) Numbers of mutations and numbers of patients with or without mutations in IS-A and IS-B groups.


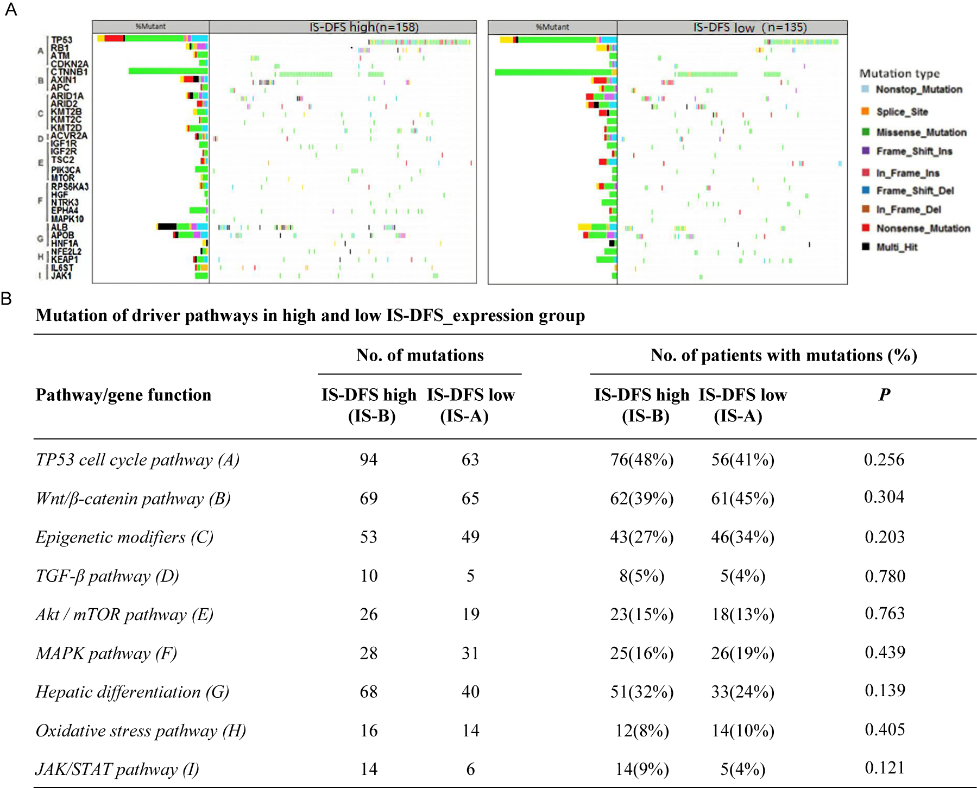


| Table S1. Antibody sources and staining conditions. | | | | |
| --- | --- | --- | --- | --- |
| Markers | Antibody source | Species | Dilution | Antigen retrieval buffer |
| CD45RO | Abcam, ab23 | Mouse monoclonal | 1:200 | Citrate buffer (pH 6.0) microwave 16min |
| CD68 | DAKO, M087601-2 | Mouse monoclonal | 1:200 | Citrate buffer (pH 6.0) microwave 16min |
| FOXP3 | Abcam, ab20034 | Mouse monoclonal | 1:100 | Citrate buffer (pH 6.0) microwave 16min |
| CD66b | Abcam, ab197678 | Rabbit monoclonal | 1:200 | Citrate buffer (pH 6.0) microwave 16min |
| CD20 | Abcam, ab9475 | Mouse monoclonal | 1:200 | Citrate buffer (pH 6.0) microwave 16min |
| TIM-3 | R&D Systems, AF2365-SP | Goat monoclonal | 1:100 | Citrate buffer (pH 6.0) microwave 16min |
| LAG3 | LifeSpan Bioscience, LS-B2237 | Mouse monoclonal | 1:100 | Citrate buffer (pH 6.0) microwave 16min |
| CTLA-4 | Santa Cruz Biotechnology, sc-376016 | Mouse monoclonal | 1:100 | Citrate buffer (pH 6.0) microwave 16min |
| PD-L1 | CST, #13684 | Rabbit monoclonal | 1:200 | Citrate buffer (pH 6.0) microwave 16min |
| PD-1 | Abcam, ab52587 | Mouse monoclonal | 1:50 | Citrate buffer (pH 6.0) microwave 16min |
| OX40 | Abcam, ab119904 | Rabbit monoclonal | 1:100 | Citrate buffer (pH 6.0) microwave 16min |

| Table S2. Clinicopathological characteristics of Patients According to the Stromal Immunotype (OS) in the TCGA Cohort. | | | |
| --- | --- | --- | --- |
| Variable |  | | |
|  | IS-low (n=184) | IS-high (n=181) | P |
| Age | 59.6 ± 13.1 | 59.5 ± 13.3 | 0.967 |
| Gender |  |  | 0.220 |
| Female | 54 (29.3%) | 64 (35.4%) |  |
| Male | 130 (70.7%) | 117 (64.6%) |  |
| T stage |  |  | 0.251 |
| I | 99 (54.4%) | 81 (45.0%) |  |
| II | 45 (24.7%) | 47 (26.1%) |  |
| III | 33 (18.1%) | 44 (24.4%) |  |
| IV | 5 (2.7%) | 8 (4.4%) |  |
| N status |  |  | 0.343 |
| Negative | 133 (99.3%) | 115 (97.5%) |  |
| Positive | 1 (0.7%) | 3 (2.5%) |  |
| Distant metastasis |  |  | 0.337 |
| No | 143 (99.3%) | 120 (97.6%) |  |
| Yes | 1 (0.7%) | 3 (2.4%) |  |
| AJCC-TNM Stage |  |  | 0.251 |
| Stage I | 94 (54.3%) | 76 (45.0%) |  |
| Stage II | 42 (24.3%) | 42 (24.9%) |  |
| Stage III | 35 (20.2%) | 48 (28.4%) |  |
| Stage IV | 2 (1.2%) | 3 (1.8%) |  |
| Tumor differentiation |  |  | 0.199 |
| Good | 121 (66.9%) | 108 (60.3%) |  |
| Poor | 60 (33.1%) | 71 (39.7%) |  |
| AJCC, American Joint Committee on Cancer. | | | |
|  |  |  |  |

| Table S3. Univariate COX Regression Analysis of 10 Immune Phenotypes and Overall Survival in the Total cohort (Testing Cohort and Validation Cohort). | | | |
| --- | --- | --- | --- |
| Variables | HR (95% CI) | P | C-index |
| **OS** |  |  |  |
| Tumor core memory T cells | 1.002 (0.999, 1.005) | 0.257 | 0.484 |
| Tumor core macrophages | 1.001 (1.000, 1.003) | 0.016 | 0.544 |
| Tumor core Tregs | 1.030 (0.916, 1.157) | 0.624 | 0.524 |
| Tumor core neutrophils | 1.001 (0.999, 1.002) | 0.263 | 0.560 |
| Stromal memory T cells | 0.998 (0.997, 0.999) | <0.001 | 0.672 |
| Stromal macrophages | 1.004 (1.003, 1.005) | <0.001 | 0.717 |
| Stromal Tregs | 1.070 (1.044, 1.095) | <0.001 | 0.673 |
| Stromal neutrophils | 1.007 (1.005, 1.009) | <0.001 | 0.681 |
| **DFS** |  |  |  |
| Tumor core memory T cells | 1.000 (0.997, 1.004) | 0.797 | 0.490 |
| Tumor core B cells | 1.000 (0.999, 1.001) | 0.703 | 0.517 |
| Tumor core Tregs | 0.983 (0.843, 1.145) | 0.823 | 0.492 |
| Tumor core neutrophils | 1.004 (1.003, 1.004) | <0.001 | 0.699 |
| Stromal memory T cells | 0.998 (0.997, 0.999) | <0.001 | 0.639 |
| Stromal B cells | 1.001 (1.001, 1.002) | <0.001 | 0.674 |
| Stromal Tregs | 1.123 (1.091, 1.156) | <0.001 | 0.666 |
| Stromal neutrophils | 1.007 (1.005, 1.008) | <0.001 | 0.653 |
| OS, overall survival; DFS, disease-free survival; Tregs, regulatory T cells; HR, hazard ratio; CI, confidence interval. | | | |

| Table S4. Univariate and multivariate analysis of the validation cohort. | | | | | | |
| --- | --- | --- | --- | --- | --- | --- |
| Variable | Univariate analysis | | | Multivariate analysis | | |
|  | HR | 95%CI | P | HR | 95%CI | P |
| **Overall survival** |  |  |  |  |  |  |
| Age | 0.99 | 0.97-1.01 | 0.455 | 1.00 | 0.976-1.024 | 0.969 |
| Gender, female vs. male | 1.02 | 0.57-1.84 | 0.949 |  |  |  |
| HBV, positive vs. negative | 1.17 | 0.59-2.30 | 0.660 | 0.65 | 0.287-1.474 | 0.303 |
| HBV-DNA, >103/≤103 IU/mL | 1.03 | 0.54-1.96 | 0.939 |  |  |  |
| AFP, ≥400 ng/mL vs. < 400 ng/mL | 1.43 | 0.85-2.40 | 0.175 |  |  |  |
| Preoperative ALT, IU/L | 1.00 | 0.99-1.01 | 0.427 |  |  |  |
| Preoperative AST, IU/L | 1.00 | 0.99-1.01 | 0.692 |  |  |  |
| ALBI Grade, Grade 2 vs. Grade 1 | 1.84 | 1.07-3.17 | 0.278 |  |  |  |
| BCLC Classification |  |  |  |  |  |  |
| BCLC-B vs. BCLC-A | 1.19 | 0.58-2.43 | 0.642 | 1.27 | 0.62-2.63 | 0.514 |
| BCLC-C vs. BCLC-A | 9.68 | 3.49-26.86 | <0.001 | 6.87 | 2.38-19.80 | <0.001 |
| Tumor differentiation, poor vs. good | 1.35 | 0.81-2.27 | 0.250 |  |  |  |
| MVI, yes vs. no | 2.36 | 1.38-4.05 | 0.002 | 1.40 | 0.73-2.69 | 0.314 |
| Immune score, IS-B vs. IS-A | 5.52 | 3.24-9.41 | <0.001 | 5.78 | 3.35-9.97 | <0.001 |
| **Disease-free survival** |  |  |  |  |  |  |
| Age | 0.98 | 0.96-1.00 | 0.075 |  |  |  |
| Gender, female vs. male | 0.45 | 0.21-0.94 | 0.035 |  |  |  |
| HBV, positive vs. negative | 1.69 | 0.76-3.73 | 0.195 |  |  |  |
| HBV-DNA, IU/mL, >103/≤103 | 1.04 | 0.55-1.98 | 0.894 |  |  |  |
| AFP, ≥400 ng/mL vs. < 400 ng/mL | 1.68 | 1.00-2.85 | 0.052 | 2.08 | 1.22-3.53 | 0.007 |
| Preoperative ALT, IU/L | 1.00 | 0.99-1.01 | 0.872 |  |  |  |
| Preoperative AST, IU/L | 1.00 | 0.99-1.01 | 0.727 |  |  |  |
| ALBI Grade, Grade 2 vs. Grade 1 | 1.26 | 0.70-2.28 | 0.447 |  |  |  |
| BCLC Classification |  |  |  |  |  |  |
| BCLC-B vs. BCLC-A | 1.66 | 0.87-3.18 | 0.124 | 1.14 | 0.54-2.41 | 0.728 |
| BCLC-C vs. BCLC-A | 3.23 | 1.13-9.27 | 0.029 | 1.39 | 0.40-4.84 | 0.608 |
| Tumor differentiation, poor vs. good | 0.83 | 0.49-1.43 | 0.511 |  |  |  |
| MVI, yes vs. no | 2.45 | 1.42-4.23 | 0.001 | 2.10 | 1.15-3.85 | 0.016 |
| Immune score, IS-B vs. IS-A | 4.35 | 2.55-7.43 | <0.001 | 4.71 | 2.70-8.21 | <0.001 |
| HBV, hepatitis B virus; AFP, alpha fetoprotein; ALT, alanine aminotransferase; AST, aspartate aminotransferase; ALBI, albumin-bilirubin; FIB-4, Fibrosis 4 Score; AJCC, American Joint Committee on Cancer; BCLC, Barcelona Clinic Liver Cancer; MVI, microvascular invasion; HR, hazard ratio; CI, confidence interval. | | | | | | |
|  |  |  |  |  |  |  |
|  |  |  |  |  |  |  |

| Table S5. Multivariable COX regression in the TCGA cohort. | | |
| --- | --- | --- |
| Variables | HR (95%CI) | P |
| Overall survival |  |  |
| TCGA cohort (N = 228)* |  |  |
| Immune score, IS-B vs. IS-A | 4.338 (1.797-10.474) | 0.001 |
| T2 vs. T1 | 1.343 (0.688-2.624) | 0.388 |
| T3 vs. T1 | 2.849 (1.622-5.002) | 0.0003 |
| T4 vs. T1 | 4.802 (2.012-11.462) | 0.0004 |
| Disease-free survival |  |  |
| TCGA cohort (N = 201)* |  |  |
| Immune score, IS-B vs. IS-A | 4.236 (1.866-9.617) | 0.0006 |
| Stage II vs. Stage I | 1.673 (1.015-2.758) | 0.044 |
| Stage III vs. Stage I | 2.647 (1.696-4.132) | <0.0001 |
| Stage IV vs. Stage I | 6.590 (1.995-21.765) | 0.002 |
| *, Age, Sex, Race, Tumor grade, Tumor stage, T stage, N stage, M stage, and AJCC-TNM stage were added in the multivariable model. HR, hazard ratio; CI, confidence interval. | | |
|  |  |  |
|  |  |  |
|  |  |  |
